# 《胸部恶性肿瘤围术期静脉血栓栓塞症预防中国专家共识（2018版）》解读之直接口服抗凝药应用前瞻篇

**DOI:** 10.3779/j.issn.1009-3419.2019.12.04

**Published:** 2019-12-20

**Authors:** 瑞恒 姜, 彤 李, 辉 李

**Affiliations:** 北京，首都医科大学附属北京朝阳医院胸外科 Department of Thoracic Surgery, Beijing Chaoyang Hospital, Capital Medical University, Beijing 100020, China

**Keywords:** 静脉血栓栓塞症, 直接口服抗凝药, 胸部恶性肿瘤, 围术期, Venous thromboembolism, Direct oral anticoagulant, Malignant tumor of chest, Perioperative period

## Abstract

胸部恶性肿瘤患者围术期静脉血栓栓塞症（venous thromboembolism, VTE）是一种需要引起重视的胸外科围术期并发症，中国胸外科静脉血栓栓塞研究协作组针对胸部恶性肿瘤患者围术期VTE的预防，发布了国际首部《胸部恶性肿瘤围术期静脉血栓栓塞症预防中国专家共识》（2018版）。本文将对直接口服抗凝药在胸部恶性肿瘤患者围术期的应用进行解读，以助于更好地理解共识相关内容。

## Abstract (en)

Venous thromboembolism (VTE) is a preventable perioperative complication of malignant tumor in thoracic surgery. At present, low molecular weight heparin anticoagulants are the first choices for perioperative drug prevention of malignant tumors, and direct oral anticoagulants are not recommended for perioperative use of malignant tumors in thoracic surgery, but their application in other related fields is relatively mature. This article will introduce direct oral anticoagulants and analyze the prospect of their perioperative application in patients with thoracic malignant tumors. It is helpful to better understand the relevant contents of "perioperative VTE prophylaxis in thoracic cancer patients: Chinese experts consensus (2018 edition)".

静脉血栓栓塞症（venous thromboembolism, VTE）是影响患者生命安全及预后的常见围术期并发症，但同时是可以预防的。2108年中国胸外科静脉血栓栓塞研究协作组发布了国际首部《胸部恶性肿瘤围术期静脉血栓栓塞症预防中国专家共识》（以下简称《共识》）。关于胸部恶性肿瘤预防VTE使用抗凝药的问题，《共识》建议首选低分子肝素，直接口服抗凝药因缺乏足够的证据支持，暂不推荐使用^[1]^。本文将介绍经典抗凝药低分子肝素（low molecular weight heparin, LMWH）应用现状和直接口服抗凝药（direct oral anticoagulant, DOAC）应用进展，并分析DOAC在胸外科恶性肿瘤围术期的应用前景，以期为DOAC应用恶性肿瘤围术期的进一步研究提供参考。

## 1 目前恶性肿瘤围术期VTE药物预防现状

在选择用药方面，经典抗凝剂低分子肝素（low-molecular-weight heparin, LMWH）是目前恶性肿瘤围术期预防性抗凝的首选用药^[2]^。LMWH具有以下优势：半衰期较短，约2 h；作用迅速，体内代谢快；抗凝疗效确切，出血事件少；引起血小板减少症少见；每天仅需皮下注射1次等。同时，LMWH存在依从性不足的缺点。最近一项包括6, 345例癌症患者的回顾性分析^[3]^发现，只有66%的患者接受并完成长期使用LMWH抗凝治疗，而其中24%的患者在第1周内转用口服抗凝药华法林，9.8%的患者在第1周后转用口服抗凝药。另一项相似的研究^[4]^结果提示，只有25%的患者接受了长期的LMWH治疗，而47.7%的患者中途改用了口服抗凝药华法林，24.1%的患者中途改用了直接口服抗凝药利伐沙班。调查发现影响LMWH依从性的主要原因是：注射部位疼痛、瘀斑、瘙痒和硬结等，担心药物潜在的副作用，包括肝素诱导的血小板减少症、过敏反应等^[5, 6]^。依从性不足的缺点又将会影响长期预防的有效性^[7]^。而直接口服抗凝药一定程度上可以改善患者依从性差的相关因素。

## 2 DOAC的应用及进展

DOAC也被称为新型（或非维生素K拮抗剂）口服抗凝药（novel oral anticoagulants, NOAC），一种是直接Xa因子抑制剂，包括利伐沙班、依度沙班、阿哌沙班等；另一种是间接Xa因子抑制剂，以达比加群酯为代表。DOAC基于其口服给药、起效迅速、剂量-疗效反应可预测、与食物和药物的相互作用小、用药期间不需监测等特点，具有广阔的用药前景^[8]^。目前，在VTE方面，DOAC已应用于骨科围术期预防性抗凝及内科恶性肿瘤患者VTE的预防和治疗。

### 2.1 DOAC在骨科围术期的应用

目前胸外科恶性肿瘤围术期尚无应用DOAC预防VTE的临床实验研究。但骨科膝、髋关节围术期应用利伐沙班的大宗研究已证明相比于经典抗凝药LMWH，利伐沙班具有更好的预防效果，并且未明显增加出血风险。一项包含3, 128例全膝关节置换术患者的多中心双盲随机对照试验中，实验组予以每日一次口服利伐沙班10 mg，对照组予以每12 h一次皮下注射依诺肝素30 mg，术前6 h-8 h即开始用药，术后12 h-24 h恢复用药。结果显示：在预防疗效上，与依诺肝素相比，利伐沙班显著降低了VTE发生的绝对风险，降低风险为3.2%（相对风险降低31%）。在安全性上，利伐沙班组有10例（0.7%）发生大出血，依诺肝素组有4例（0.3%）发生大出血（*P*=0.109, 6）；利伐沙班组有46例（3.0%）发生临床相关的非大出血，依诺肝素组有34例（2.3%）发生临床相关的非大出血（*P*=0.179, 0）。与依诺肝素相比，尽管利伐沙班组总出血事件发生率偏高，但两者间没有统计学差异^[9]^。另一项关于全膝关节置换术围术期应用利伐沙班预防VTE的试验^[10]^结果显示，与应用依诺肝素（40 mg, *qd*）相比，应用利伐沙班（10 mg, *qd*）显著降低了VTE的发生率，且大出血事件的发生率更低。当利伐沙班用于髋关节置换术围术期的延长预防时，依然表现出优于依诺肝素的预防效果，且二者安全性相当^[11]^。2011年一项关于利伐沙班应用于膝、髋关节置换术围术期预防VTE的荟萃分析^[12]^表明：与依诺肝素相比，利伐沙班组出血事件有一定的增加，但并没有统计学差异。国内也有相关研究^[13]^表明，利伐沙班是应用于膝、髋关节置换术围术期预防性抗凝更方便、更安全的药物。在2012年的第9版美国胸内科医师学会（American College of Chest Physicians, ACCP）指南中，已明确推荐利伐沙班可用于膝、髋关节置换术围术期VTE的预防^[14]^。可见，利伐沙班在全膝关节置换术围术期的使用是安全有效的，其口服给药的方式优于LMWH的皮下注射，因此在骨科围术期已得到广泛的应用。

### 2.2 DOAC在内科恶性肿瘤患者VTE预防、治疗中的应用

与非恶性肿瘤患者相比，恶性肿瘤患者更易患VTE，相关研究^[15]^指出恶性肿瘤是VTE的独立危险因素。恶性肿瘤患者经常患有多种合并症，与非恶性肿瘤患者相比，一旦发生VTE，预后相对较差，且治疗过程中出血和VTE复发的风险均更大。一直以来，长期使用LMWH是治疗恶性肿瘤患者VTE的标准治疗方式^[16-18]^。而DOAC的出现也提供了一种新的抗凝治疗选择。一项包含1, 050例有急性症状或偶发VTE的恶性肿瘤患者的研究^[19]^中，实验组先给予至少5 d的LMWH治疗，后续给予依度沙班6个月-12个月的抗凝治疗，对照组使用LMWH抗凝治疗6个月-12个月。结果显示：实验组的VTE复发率明显低于对照组（分别为7.9%和11.3%；*P*=0.09），但实验组大出血事件的发生率高于对照组（分别为6.9%和4.0%；*P*=0.04），而两组严重大出血事件的发生率相似。因此，就VTE复发率和大出血发生率而言，依度沙班的治疗效果并不逊于LMWH。另一项比较利伐沙班和达肝素钠预防恶性肿瘤患者VTE的随机对照试验^[20]^结果显示，利伐沙班组的VTE复发率显著低于LMWH组（分别为11%和4%），大出血风险则略高于LMWH组（分别为6%和4%）。值得注意的是，利伐沙班组的食管恶性肿瘤和贲门恶性肿瘤患者发生大出血的风险显著偏高，且主要发生于消化道。因此对于消化道恶性肿瘤患者，使用直接口服抗凝药需谨慎。除利伐沙班、依度沙班外，直接口服抗凝药如阿哌沙班等在VTE的治疗和预防复发中也有良好的效果^[21, 22]^。

基于DOAC在内科恶性肿瘤患者VTE的治疗上表现出可靠的安全性和更佳的有效性，其在恶性肿瘤患者VTE预防方面的研究也相继报道。一项包含574例恶性肿瘤化疗患者预防VTE的研究，在利用Khorana评分筛选中高危VTE患者后，实验组予以阿哌沙班2.5 mg每天2次预防抗凝6个月，对照组予以安慰剂治疗。结果显示：实验组VTE发生率显著低于安慰剂组（分别为4.2%和10.2%，*P* < 0.001），阿哌沙班组大出血事件发生率则高于安慰剂组（分别为3.5%和1.8%，*P*=0.046）。值得注意的是，大出血事件主要发生于胃肠道恶性肿瘤患者和妇科恶性肿瘤患者，因此DOAC应用于此类患者时应当谨慎^[23]^。最新的一项关于利伐沙班用于门诊恶性肿瘤高危患者VTE预防的研究^[24]^指出：有效性上，利伐沙班组VTE发生率为6.0%，低于安慰剂组的8.8%（*P*=0.10）；安全性上，利伐沙班组大出血事件发生率为2%，高于安慰剂组的1%。一项荟萃分析^[25]^结果显示，与LMWH相比，DOAC更能降低恶性肿瘤患者VTE发生率，且DOAC的治疗时长通常长于LMWH，间接表明了患者对DOAC的偏爱及更好的依从性。最近国际血栓与止血学会（International Society on Thrombosis and Haemostasis, ISTH）也发布了关于肿瘤相关性血栓（cancer-associated thrombosis, CAT）的指南，推荐使用依度沙班和利伐沙班等直接口服抗凝药用于确诊VTE的癌症患者^[26]^。美国临床肿瘤学会（American Society of Clinical Oncology, ASCO）指南同样提出，利伐沙班、依度沙班可以用于门诊VTE高危的恶性肿瘤患者进行预防性抗凝^[18]^。

## 3 总结

《共识》暂不推荐利伐沙班等直接口服抗凝药应用于胸部恶性肿瘤围术期VTE的预防，其主要原因是目前尚无相关研究证实DOAC在恶性肿瘤尤其是胸部恶性肿瘤围术期应用的安全性和有效性。但目前，DOAC已广泛应用于骨科围术期、内科恶性肿瘤等VTE的预防或治疗，在安全性和有效性方面已得到充分的验证，并且与LMWH相比，DOAC能提高患者的依从性。因此，我们相信DOAC应用于胸外科围术期VTE预防是值得期待和尝试的，同时相关研究中也指出食管及胃肠道等消化道恶性肿瘤患者出血风险高，因此对于此类患者应用DOAC时应当谨慎。
